# Innate immune activating ligand SUMOylation affects tumor cell recognition by NK cells

**DOI:** 10.1038/s41598-017-10403-0

**Published:** 2017-09-05

**Authors:** Beatrice Zitti, Rosa Molfetta, Cinzia Fionda, Linda Quatrini, Helena Stabile, Mario Lecce, Valeria de Turris, Maria Rosaria Ricciardi, Maria Teresa Petrucci, Marco Cippitelli, Angela Gismondi, Angela Santoni, Rossella Paolini

**Affiliations:** 1 Department of Molecular Medicine, “Sapienza” University of Rome, Laboratory affiliated to Istituto Pasteur Italia - Fondazione Cenci Bolognetti, “Viale Regina Elena 291, 00161 Rome, Italy; 2 Center for Life Nanoscience, Istituto Italiano di Tecnologia, Rome, Italy; 3 Division of Hematology, Department of Clinical and Molecular Medicine, Sapienza University of Rome, Rome, Italy; 4 Department of Cellular Biotechnologies and Hematology, Sapienza University of Rome, Rome, Italy; 5 Istituto Mediterraneo di Neuroscienze, Neuromed, Pozzilli Italy; 6 Centre d’Immunologie de Marseille-Luminy, Aix Marseille Université UM2, Inserm, U1104, CNRS UMR7280, 13288 Marseille, France

## Abstract

Natural Killer cells are innate lymphocytes involved in tumor immunosurveillance. They express activating receptors able to recognize self-molecules poorly expressed on healthy cells but up-regulated upon stress conditions, including transformation. Regulation of ligand expression in tumor cells mainly relays on transcriptional mechanisms, while the involvement of ubiquitin or ubiquitin-like modifiers remains largely unexplored. Here, we focused on the SUMO pathway and demonstrated that the ligand of DNAM1 activating receptor, PVR, undergoes SUMOylation in multiple myeloma. Concurrently, we found that PVR is preferentially located in intracellular compartments in human multiple myeloma cell lines and malignant plasma cells and that inhibition of the SUMO pathway promotes its translocation to the cell surface, increasing tumor cell susceptibility to NK cell-mediated cytolysis. Our findings provide the first evidence of an innate immune activating ligand regulated by SUMOylation, and confer to this modification a novel role in impairing recognition and killing of tumor cells.

## Introduction

Natural Killer (NK) cells represent a subset of innate lymphocytes largely involved in tumor immunosurveillance because of their ability to recognize and kill transformed cells and to secrete cytokines and chemokines^1, 2^. Their activation is controlled by the integration of signals induced by inhibitory receptors, which recognize Major Histocompatibility Complex (MHC) class I molecules on healthy cells, and activating receptors able to bind ligands up-regulated in stressed cells^3^.

Therefore, understanding the molecular mechanisms underlying the expression of NK cell activating ligands on tumor cells is crucial for the development of new therapeutic anti-cancer approaches aimed at improving NK cell-mediated tumor clearance.

Several lines of evidence^4–8^ have reported a pivotal role for NK cells in controlling the progression of Multiple Myeloma (MM), an incurable age-dependent haematological neoplasia characterized by abnormal proliferation of malignant plasma cells (PCs) in the bone marrow (BM), associated with serum monoclonal gammopathy, bone destruction, and several organ dysfunctions^9–11^. Indeed, NK cells increase in number at the initial stages of the MM disease contributing to limit malignant PC expansion, while tumor progression is associated with a decline of NK cell surveillance^4–8^.

Different activating receptors are involved in NK cell-mediated MM cell recognition and elimination, after engaging of their ligands^12–14^. In particular, several studies have demonstrated that the interaction of the activating NK cell receptor DNAX accessory molecule 1 (DNAM1/CD226) with its ligands, Nectin2 (CD112) and Poliovirus Receptor (PVR/CD155)^15, 16^, contributes to the killing of MM cells^13, 14^. Moreover, a pivotal role played by DNAM1 in the control of tumor growth was reported in an *in vivo* model of spontaneous MM progression^15, 17^.

Although expressed on normal cells including neuronal, epithelial, endothelial and fibroblastic cells, Nectin2 and PVR are found up-regulated on tumor cells^14, 18–21^. Several studies have documented transcriptional regulation of DNAM1 ligand expression in response to different stimuli^14, 22–25^, while the involvement of post-translational mechanisms has been poorly investigated so far.

We focused on the SUMO pathway, an emerging post-translational modification that was found deregulated in many tumors, including breast and lung cancer, glioblastoma and MM^26–28^. It is catalyzed by the sequential action of three classes of enzymes, namely E1, E2 and E3, and culminates in the covalent addition of a member of the SUMO (small ubiquitin-like modifier) protein family to lysine residues of specific targets^29, 30^. SUMO modification leads to different outcomes: it can affect the enzymatic activity of target proteins, their ability to interact with other macromolecules as well as their subcellular localization^29, 30^.

It is well documented that under stress conditions, including malignant transformation, a general increase in protein SUMO conjugation occurs^30^, often as a result of the E2 SUMO conjugating enzyme UBC9 overexpression^26–28, 31, 32^. In particular, overexpression of UBC9 and of other SUMO pathway components in MM cells correlates with poor prognosis^28^. However, whether the SUMO pathway affects tumor recognition by immune cells is currently unknown.

To gain insight into this issue we investigated whether the SUMO pathway regulates PVR and Nectin2 expression on MM cells.

Here, we show that both DNAM1 ligands are expressed in MM cell lines and patient’s derived malignant plasma cells (PC), and preferentially localized in intracellular compartments. The SUMO pathway controls PVR, but not Nectin2 surface expression. PVR is directly subjected to SUMOylation and this modification prevents its surface expression impairing DNAM1-mediated NK cell recognition.

We have also provided evidence that the SUMO pathway regulates PVR surface expression in tumors other than MM, supporting a more general role for this modification in regulating tumor cell susceptibility to NK cell-mediated cytotoxicity.

These data reveal a previously unknown role for the SUMO pathway and provide novel insights in molecular mechanisms underlying expression of innate immune activating ligands on tumor cells.

## Results

### The SUMO pathway regulates PVR but not Nectin2 cell surface expression in MM cells

**Figure 1:**
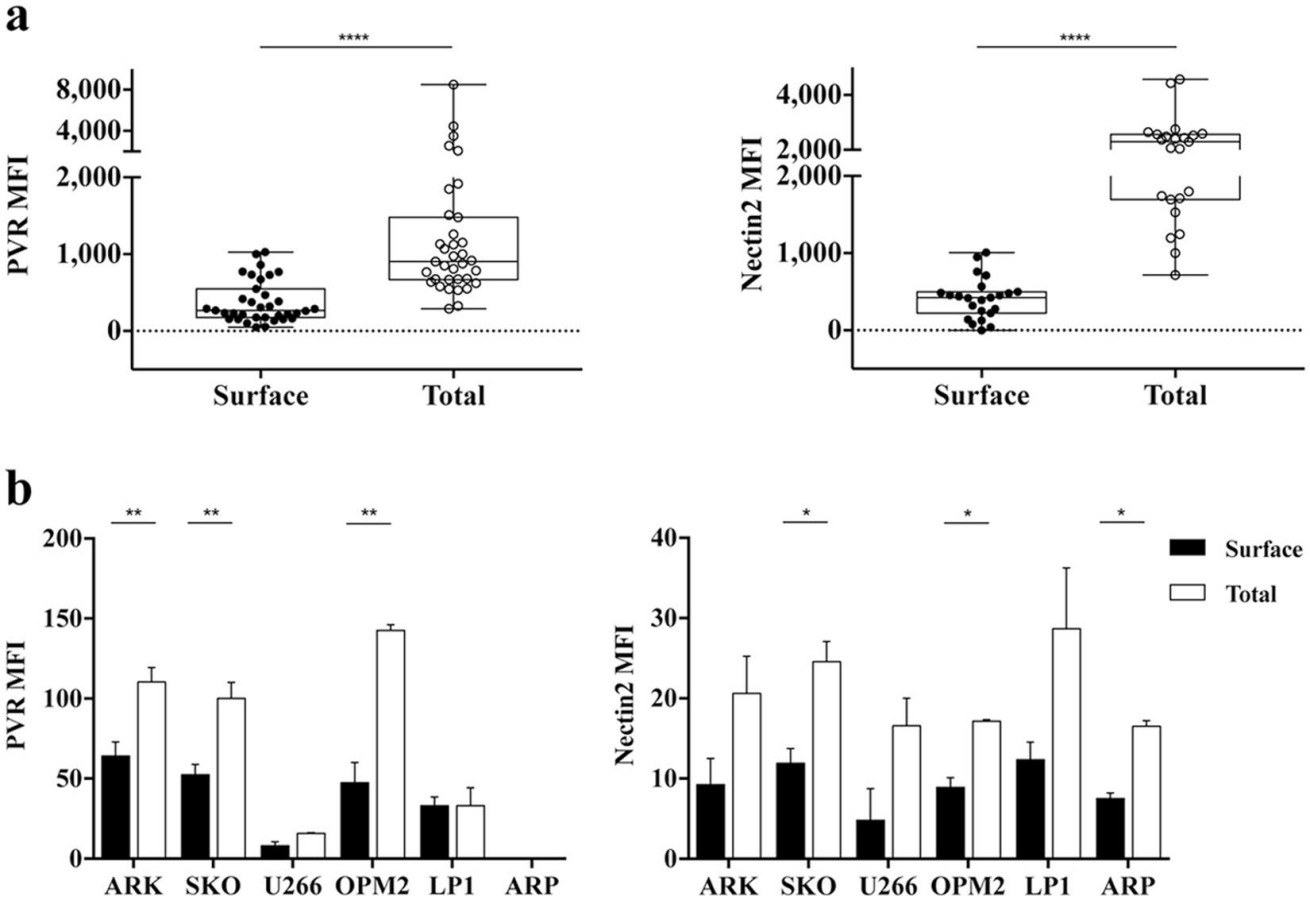
PVR and Nectin2 are mainly found as intracellular pool in MM cells. (**a**) CD38^+^ CD138^+^ malignant PCs derived from BM aspirates of MM patients (n = 34) were analysed for PVR and Nectin2 surface and total (surface plus intracellular) expression before and after fixation and permeabilization, respectively. Cells were acquired using FACSCanto flow cytometer (BD Biosciences). Each dot represents a single patient, ****p < 0.001, Wilcoxon matched pairs test. (**b**) PVR (left panel) and Nectin2 (right panel) surface and total (surface plus intracellular) expression was analysed on MM cell lines before and after fixation and permeabilization, respectively. Cells were acquired using FACSCalibur flow cytometer (BD Biosciences). Data represent the means ± SD of PVR and Nectin2 from three independent experiments. *p < 0.05, Student T test. MFI: mean fluorescence intensity.

To investigate whether post-translational mechanisms are involved in PVR and Nectin2 expression in MM cells, we initially evaluated surface and total (surface plus intracellular) protein levels by immunofluorescence and FACS analysis on malignant PCs derived from MM patients at different clinical stages (Supplementary Table 1) before and after cell permeabilization, respectively. After excluding doublets, the analysis was restricted to CD38^+^CD138^+^ malignant PCs (Supplementary Fig. S1a). PVR and Nectin2 protein expression was always much higher upon cell permeabilization with respect to unpermeabilized cells (Fig. 1a), independently from the clinical stage of the disease (Supplementary Fig. S1b). These results indicate that a pool of both proteins is present in intracellular compartments. As negative control, we verified that circulating B cells from healthy donors are negative for both surface and total DNAM1 ligand expression (data not shown).

Next, we analysed a panel of human MM cell lines and demonstrated that total expression levels of both PVR and Nectin2 were higher than their surface levels in the vast majority of them (Fig. 1b).

Altogether, these results suggest the involvement of post-translational mechanisms in the regulation of PVR and Nectin2 surface expression.

To investigate whether the SUMO pathway could be responsible for the intracellular retention of PVR and Nectin2 in MM cells, we took advantage of the use of Ginkgolic Acid (GA), an inhibitor of SAE1/UBA2, the E1 enzyme responsible for SUMO modification^33^.

**Figure 2:**
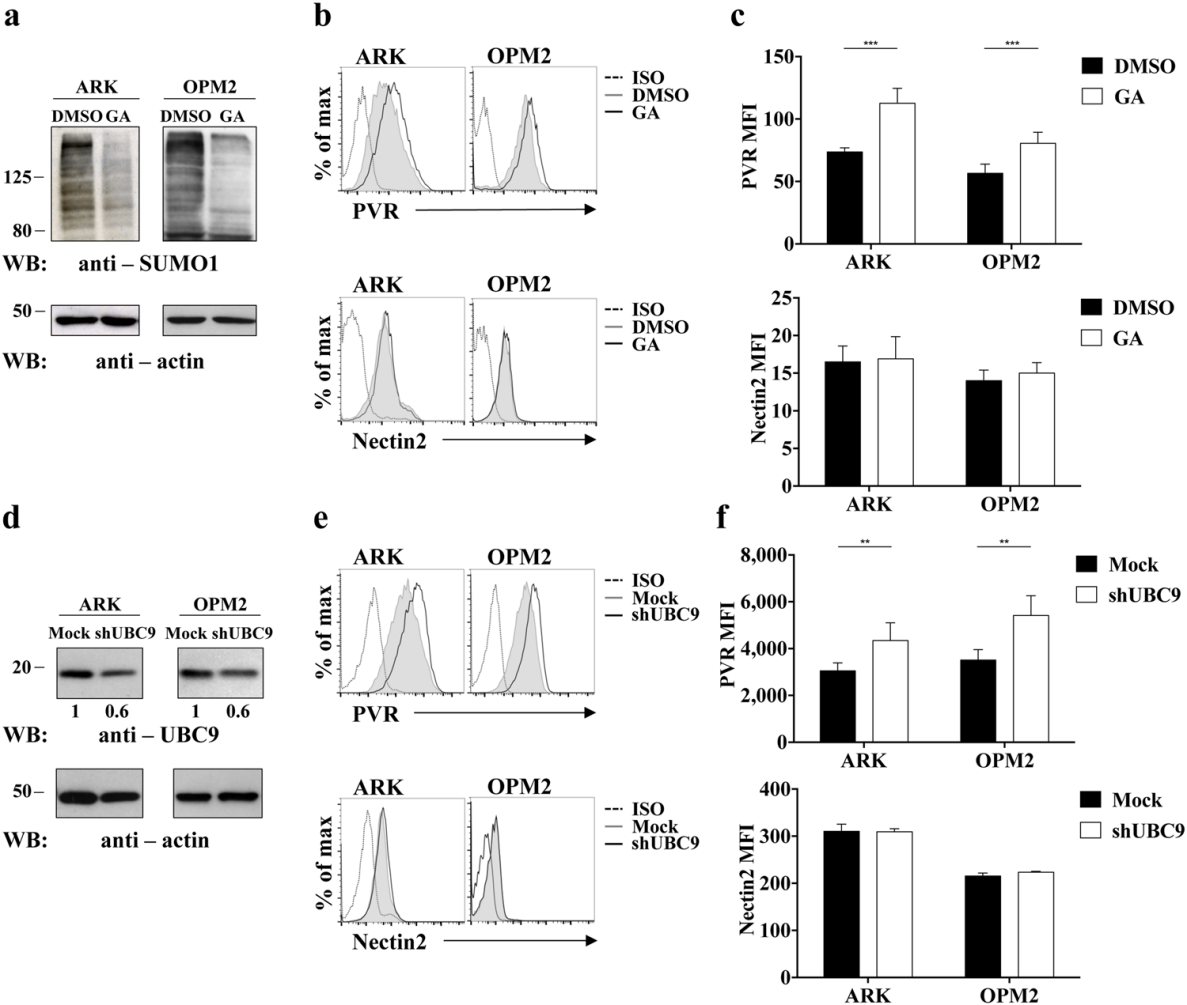
SUMOylation controls PVR but not Nectin2 surface expression in MM cell lines. (**a–c**) Inhibition of the SUMO pathway was achieved by means of overnight treatment with 25μg/mL Ginkgolic Acid (GA). The efficacy of treatment was verified by means of western blot analysis on total cell lysates (**a**). (**b**,**c**) PVR and Nectin2 surface expression was evaluated on ARK and OPM2 cell lines by immunofluorescence and FACS analysis using FACSCalibur flow cytometer (BD Biosciences). One out of three independent experiments (**b**) and means ± SD of PVR and Nectin2 MFI from three independent experiments (**c**) are shown. **p < 0.01, ***p < 0.001, Two-way ANOVA. (**d–f**) Inhibition of the SUMO pathway was achieved by means of UBC9 gene silencing. Silencing efficiency was verified by means of western blot analysis on total cell lysates (**d**). (**e**,**f**) PVR and Nectin2 surface expression was evaluated on ARK and OPM2 cell lines by immunofluorescence and FACS analysis using FACSCanto flow cytometer (BD Biosciences). One out of three independent experiments (**e**) and means ± SD of PVR and Nectin2 MFI from three independent experiments (**f**) are shown. **p < 0.01, ***p < 0.001, Two-way ANOVA.

ARK and OPM2 cell lines were treated overnight with GA, and the treatment efficacy was verified by means of a reduced amount of SUMOylated proteins on cell lysates (Fig. 2a). Upon GA treatment, we found that PVR surface expression was enhanced in both cell lines (Fig. 2b and c, upper panels), while the expression of Nectin2 (Fig. 2b and c, lower panels) and that of MHC-I used as negative control (data not shown) remained unaffected.

To further confirm these data with a different approach, we stably infected ARK and OPM2 cells lines with a lentiviral vector carrying a shRNA that targets the E2 SUMO conjugating enzyme UBC9^34^. Although we only obtained around 40% of UBC9 protein reduction (Fig. 2d), UBC9 knockdown resulted in an increased of PVR but not Nectin2 surface expression in both ARK and OPM2 cell lines (Fig. 2e and f), confirming the results obtained upon GA treatment.

**Figure 3:**
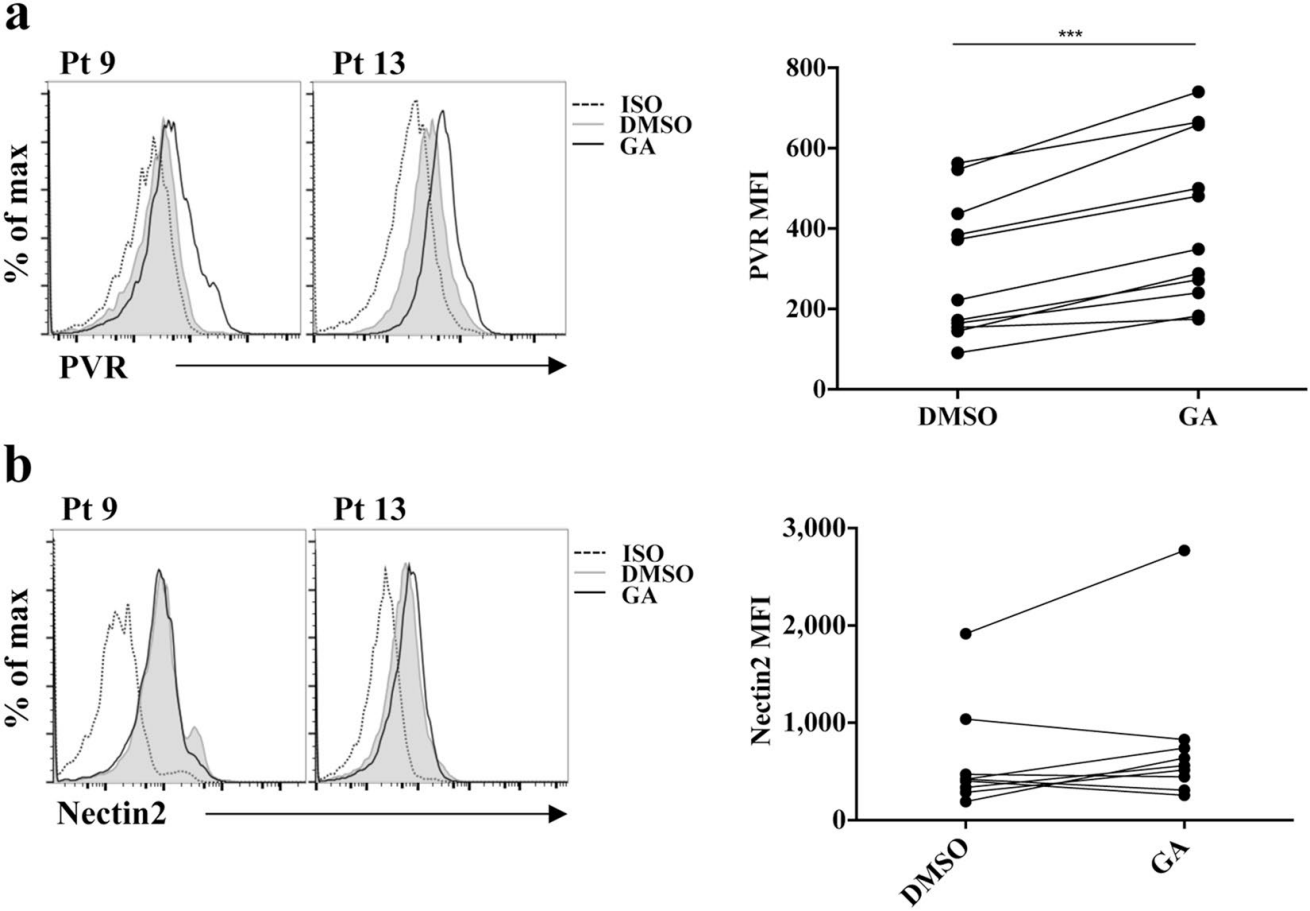
Ginkgolic Acid treatment up-regulates PVR but not Nectin2 surface expression in malignant PCs. Malignant PCs were treated overnight with 25 μg/mL of GA or vehicle alone (DMSO), and PVR (**a**) and Nectin2 (**b**) surface expression was evaluated on cells gated as in Supplementary Fig. 1a. Data from two representative patients are shown in left panels. Data from 9 patients analysed are shown in right panels. Each dot represents a single patient. ***p < 0.001 Wilcoxon matched pairs test.

We then analysed the involvement of SUMO pathway in the regulation of PVR expression on patient malignant PCs. Upon GA treatment, we observed a significant and reproducible increase in PVR but not Nectin2 surface expression in most of the MM patients analysed (Fig. 3a and b).

Altogether these results support the conclusion that the SUMO pathway regulates PVR expression in MM cells.

### PVR is a target of SUMOylation

**Figure 4:**
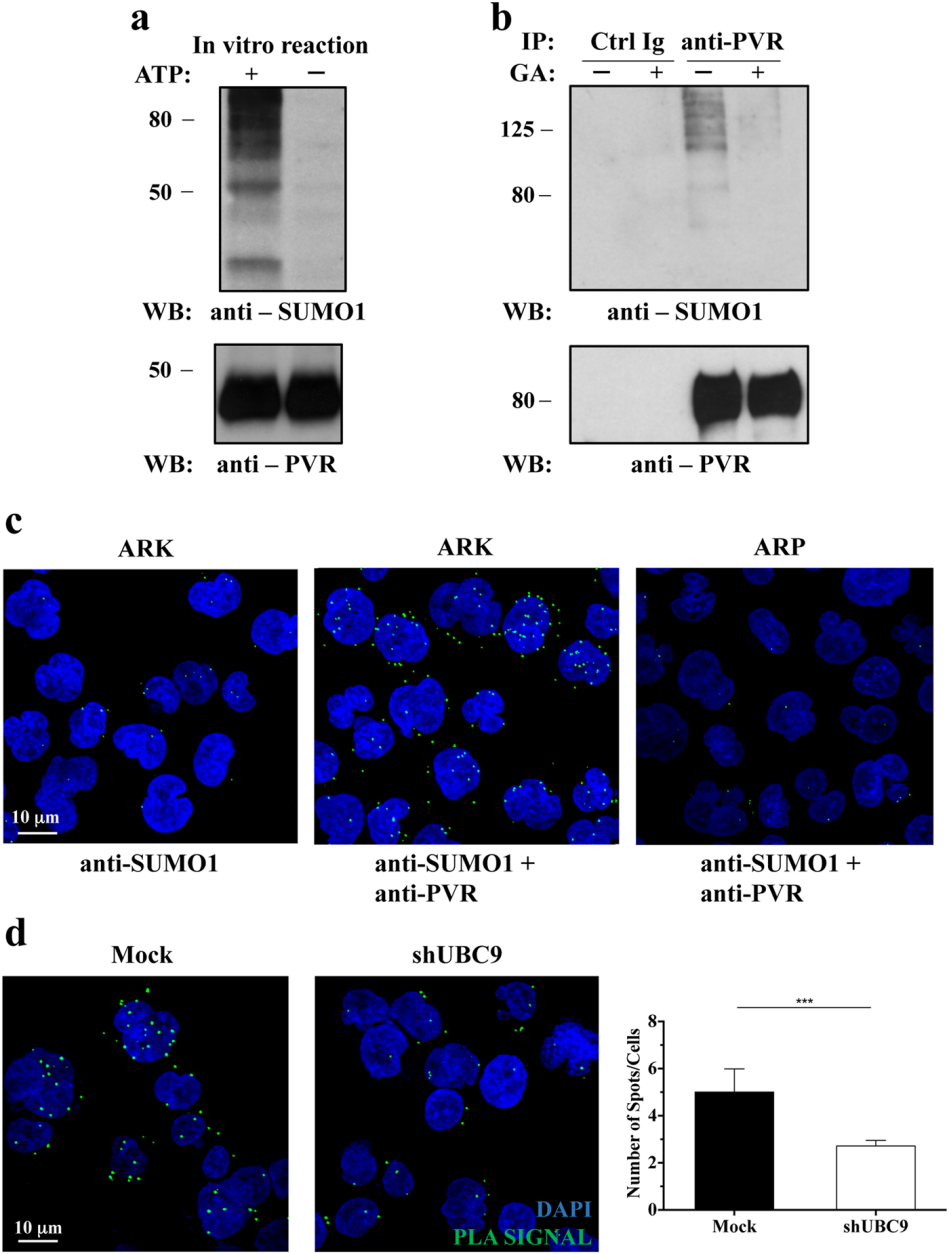
PVR undergoes SUMOylation in MM cells. (**a**) Human recombinant full length PVR was used as substrate for *in vitro* SUMOylation assay. The reaction was carried out in the presence (+) or in the absence (−) of ATP. The presence of modified PVR species was evaluated by western blot analysis using anti-SUMO1 (Y299) and anti-PVR (S-18) Abs. (**b**) ARK cells were treated (+) or not (−) over-night with 25 μg/mL of GA, and then lysed. Cell lysates were immunoprecipitated with anti-PVR (SKII.4) or with an isotype-matched Ab (Ctrl Ig). Immunoprecipitated proteins were split and separated in two SDS/PAGE, transferred on nitrocellulose filters, and immunoblotted as indicated. (**c**) Proximity ligation assay (PLA) was performed on ARK and ARP cell lines. Anti-PVR (D171) and anti-SUMO1 (BML-PW9460) were used as primary Abs, as indicated. Positive PLA signals are visualized as green fluorescent spots, nuclei are in blue (DAPI). Images are representative of three independent experiments, and were acquired with zoom 2 using 60X/1.35NA oil immersion objective. Z-projections of 40 slices are shown. (**d**) Proximity ligation assay was performed and analysed as in Fig. 4c on UBC9-silenced or Mock infected ARK cells (left panels). Number of spots per cell was quantified using Fiji ImageJ Software (right panel). Data represent the means ± SD of three independent experiments. **p < 0.01, Student T test.

To elucidate if PVR can be directly modified by SUMO, we performed an *in vitro* SUMOylation assay by using as a substrate a human recombinant full-length form of PVR. The addition of ATP in the presence of SUMO1 protein and the enzymes involved in the reaction, promoted the appearance of SUMO1 conjugates of high molecular weight (MW) that are specifically recognized by anti-SUMO1 antibody, indicating that PVR can be subjected to SUMOylation (Fig. 4a).

To further investigate whether PVR undergoes SUMOylation in MM cells, PVR was immunoprecipitated from lysates of ARK cells treated or not with GA (Fig. 4b). In untreated cells, anti-SUMO1 immunoblot revealed the presence of several bands of high MW that disappeared upon GA treatment, suggesting that they correspond to SUMOylated PVR species.

To formally demonstrate that PVR is directly SUMOylated in MM cells, we exploited the approach of Proximity Ligation Assay (PLA) that allows *in situ* detection of protein interactions and modifications with high specificity and sensitivity; ARK cells were incubated with anti-SUMO1 alone (as negative control) or in combination with anti-PVR, processed following PLA protocol, and analysed by confocal microscopy. We detected specific green fluorescent spots only in ARK cells stained with both anti-PVR and anti-SUMO1 antibodies, demonstrating that PVR directly interacts with SUMO1 (Fig. 4c, middle panel). The same PLA reaction performed in a different cell line (ARP) that does not express PVR (see Fig. 1b), did not generate any significant fluorescent spots (Fig. 4c, right panel), confirming the specificity of this assay. A 3D reconstruction of all optical slices acquired along z-axis (Supplementary Multimedia File 1) confirmed the cytoplasmic localization of PLA spots.

PLA assay was also carried out on ARK cells stably transfected with UBC9-shRNA or with Ctrl-shRNA (Mock). We observed a significant reduction of PLA spots upon UBC9 silencing (Fig. 4d), as confirmed by quantification analysis, indicating that the inhibition of SUMO pathway strongly decreases the amount of SUMOylated PVR.

Altogether our data demonstrate that PVR undergoes SUMOylation in MM cells.

### SUMOylation of PVR controls its subcellular localization

**Figure 5:**
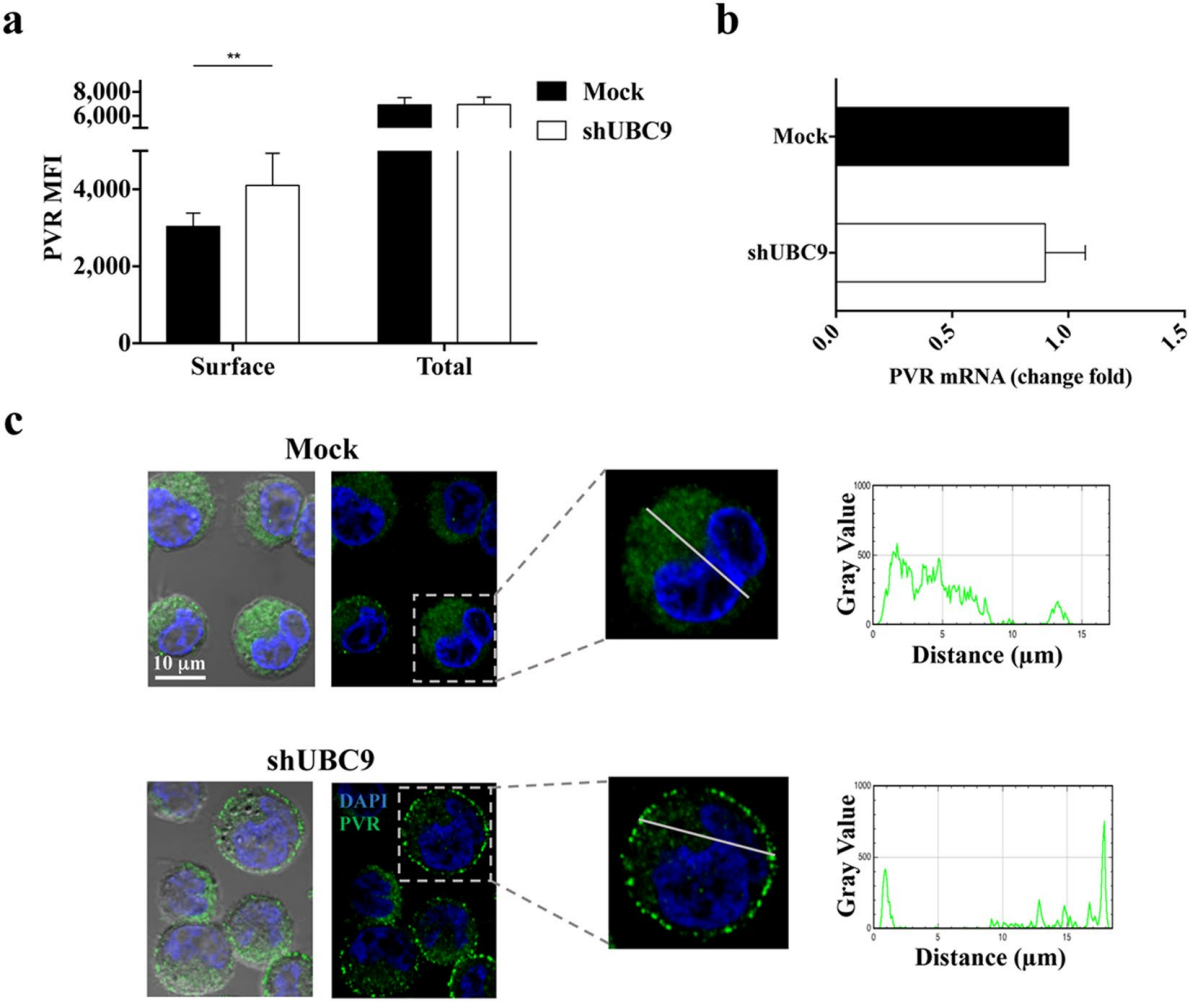
PVR subcellular localization is controlled by SUMOylation. (**a**) PVR surface and total expression was analysed on ARK cells upon UBC9 gene silencing as in Fig. 1b. Cells were acquired using FACSCanto flow cytometer. Data represent the means ± SD of three independent experiments. **p < 0.005, Two-way RM ANOVA. (**b**) Total RNA, extracted from Mock or UBC9 silenced ARK cells (shUBC9) was used for cDNA first-strand synthesis, and Real-time polymerase chain reaction for PVR mRNA was performed using the ABI Prism 7900 Sequence Detection system. Relative mRNA amount, normalized with GAPDH, was expressed as arbitrary units and referred to mock-transfected cells, considered as calibrator. Means ± SD of three independent experiments are shown. (**c**) Mock infected and UBC9-silenced ARK cells were stained with anti-PVR mAb (clone D171) followed by Alexa 488-conjugated goat anti-mouse Ab and counterstained with DAPI. Fluorescence and Differential Interference Contrast images were acquired with zoom3 using 60X/1.35NA oil immersion objective. A single optical slice (left panels) and a plot of pixel intensity of PVR signal along a section of interest (right panels) are shown.

To investigate the role of PVR SUMOylation, surface and total PVR protein expression was initially analysed by flow cytometry before and after inhibition of the SUMO pathway. Upon UBC9 silencing (Fig. 5a) or GA treatment (Fig. S2a), we observed that the total protein expression of PVR remains unaffected compared to control cells, despite the increase of protein levels at the cell surface. As expected, quantitative real time PCR performed on ARK cells revealed that PVR mRNA amount does not change in UBC9 silenced cells respect to control samples (Fig. 5b), thus excluding a regulation occurring at transcriptional level. Similar results were obtained upon GA treatment (Fig. S2b).

Finally, we analysed PVR subcellular localization by confocal microscopy. We observed that PVR is mainly distributed throughout the cytoplasm in control cells, while it is preferentially localized at the plasma membrane upon inhibition of the SUMO pathway (Figs 5c and S2c).

Altogether these results demonstrate that the inhibition of the SUMO pathway promotes the redistribution of the intracellular pool of PVR, resulting in its up-regulation on the cell surface without any effect on total protein expression.

### Inhibition of the SUMO pathway increases MM susceptibility to NK cell mediated lysis

**Figure 6:**
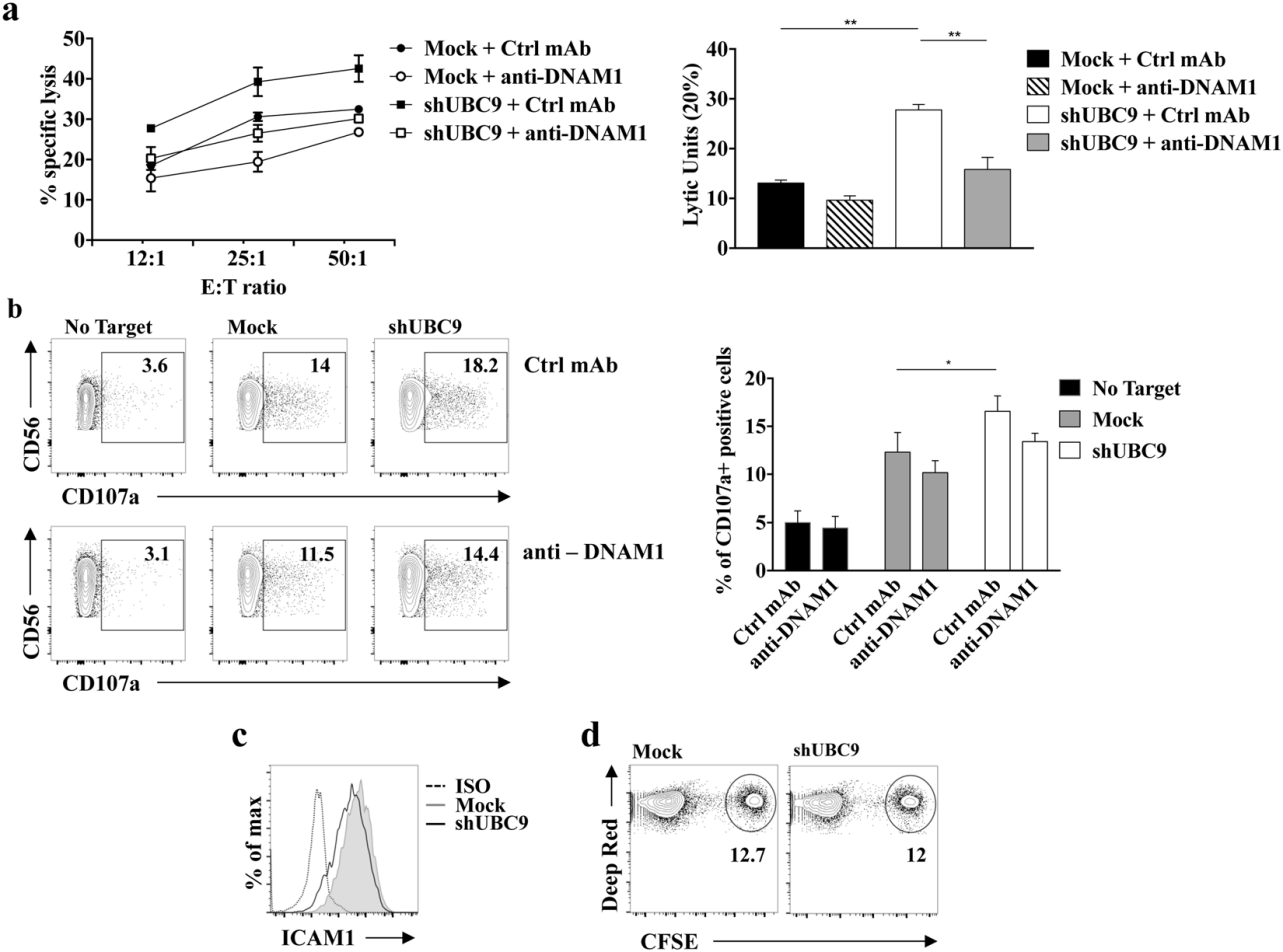
Inhibition of PVR SUMOylation increases MM cell susceptibility to NK cell mediated lysis. Primary cultured NK cells were pre-treated for 20 minutes at RT with anti-DNAM1 neutralizing mAb or with anti-CD56 (Ctrl mAb) and were used as effector cells in a 4 hours ^51^Cr release assay (**a**) or in a CD107a degranulation assay (**b**) toward shUBC9 infected or mock infected cells. (**a**) NK cells were incubated with target cells at the indicated E:T ratios. The percentage of specific lysis (left panel) and Lytic Units (right panel), calculated as described in Materials and Methods, from three independent experiments (mean ± SD) are shown. *p < 0.05, **p < 0.01, One-way ANOVA. (**b**) After 4 hours of co-culture cells were stained with CD107a-APC, CD56-PE, CD3-FITC and CD138-PerCP-CY5.5 to gate the CD56^+^ CD3^−^ CD138^−^ NK cell population, and the analysis was performed with FACSCanto (BD Biosciences). Left panels: the percentage of CD107a^+^ NK cells is shown and is representative of three independent experiments. Right panel: Means ± SD of three independent experiments are shown. *p < 0.05, **p < 0.01, Two-way ANOVA. (**c**) ICAM1 expression on UBC9 silenced or Mock infected ARK cells was measured by immunofluorescence and FACS analysis. One representative experiment out of three is shown. (**d**) Primary cultured NK cells were labelled with CellTracker Deep Red (TermoFisher), and then let to interact for 30 minutes with Mock infected or UBC9 silenced ARK cells labelled with CFSE. The percentage of the double positive cells is shown.

To investigate whether the inhibition of the SUMO pathway affects MM susceptibility to NK cell-mediated killing, we performed a cytotoxicity assay in which primary cultured human NK cells were used as effector cells. UBC9-silenced ARK cells were lysed more efficiently respect to control cells at all effector:target (E:T) ratios analysed (Fig. 6a). Moreover, we observed that in the presence of anti-DNAM1 blocking antibody NK cell cytotoxicity towards UBC9 silenced target cells is more extensively inhibited compared to Mock target cells (45% and 25% of inhibition, respectively), strongly suggesting that PVR surface up-regulation contributes to the increased susceptibility to NK cell lysis. Similar results were obtained using as targets GA-treated ARK cells (Fig. S2d) but not GA-treated LP1 cells (Fig. S2e), a MM cell line that does not show PVR intracellular retention (see Fig. 1b).

Inhibition of the SUMO pathway also resulted in enhanced ability of MM cells to stimulate NK cell degranulation measured by means of membrane expression of the cytotoxic granule marker CD107a (Fig. 6b).

The partial inhibition of both cytotoxicity and degranulation observed in the presence of anti-DNAM1 blocking antibody may be explained by the involvement of a receptor other than DNAM1.

Besides DNAM1, a major role for NKG2D activating receptor on MM cell killing has been also established^13, 14^. However, a contribution of NKG2D/NKG2D ligand axis in our experimental setting can be excluded since ARK cells do not express the NKG2D ligands MIC and ULBP, before and after inhibition of SUMOylation (Supplementary Fig. S3).

It has been shown that DNAM1 activation requires the association with the adhesion molecule LFA1^35^, therefore we checked the expression levels of the LFA1 ligand, ICAM1, on MM cells. Upon inhibition of the SUMO pathway, FACS analysis demonstrated that ICAM1 expression was not significantly affected compared to control cells (Fig. 6c), excluding the possibility that the enhanced NK cell degranulation and killing may depend on an increased LFA1/ICAM1 interaction. Moreover, we found that the number of conjugates between NK and ARK cells before and after UBC9 silencing does not change in the same experimental setting (Fig. 6d).

Altogether these results support the conclusion that the enhanced susceptibility to NK cell killing mostly depends on the increased surface expression of PVR.

**Figure 7:**
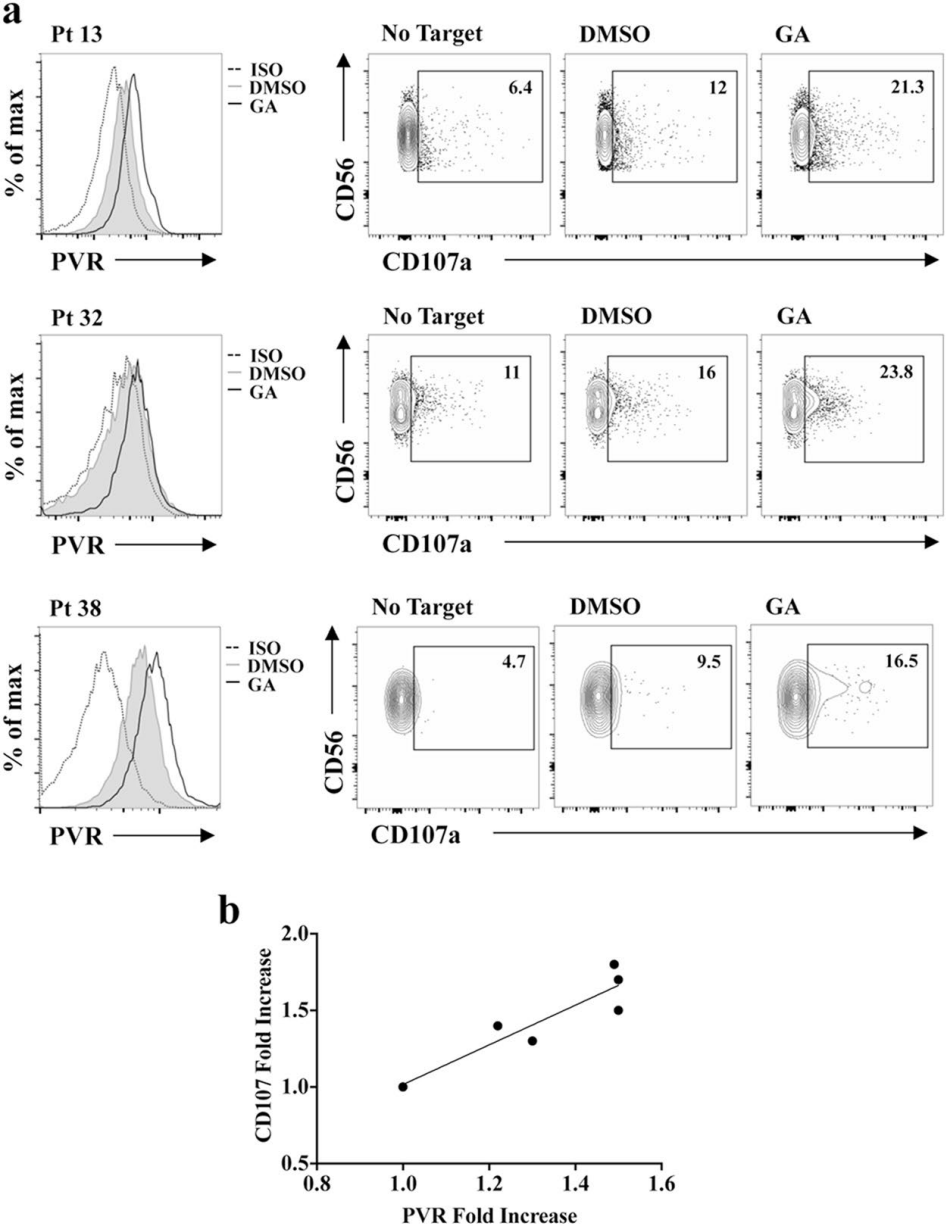
Inhibition of PVR SUMOylation in patient-derived malignant PCs enhances autologous NK cell degranulation. NK cells derived from BM aspirates of MM patients were used as effector cells in a CD107a degranulation assay toward CD38^+^ CD138^+^ autologous MM cells treated overnight with GA or vehicle alone (DMSO). After 2 hours of co-culture cells were stained and analysed as in Fig. 6b. (**a**) Data from three representative patients are shown. (**b**) Correlation between PVR fold increase on MM cells and fold increase of NK cell autologous degranulation is shown (R = 0.83; p < 0.05).

Accordingly, highly purified patient derived CD138^+^ PCs treated with GA induced a higher extent of autologous BM-derived NK cell degranulation compared to vehicle-treated cells (Fig. 7a). Noteworthy, the fold induction of NK cell degranulation directly correlated with the increase of PVR surface expression on CD138^+^ MM cells (Fig. 7b).

Collectively, our data demonstrate that PVR SUMOylation impairs the ability of NK cells to recognize and kill MM cells in a DNAM1-dependent manner.

### PVR SUMOylation affects susceptibility to NK cell lysis in tumors different from MM

Up-regulation of the SUMO pathway is a common feature of several types of cancer^26, 27^, thus we decided to extend our analysis on other high malignant tumors.

**Figure 8:**
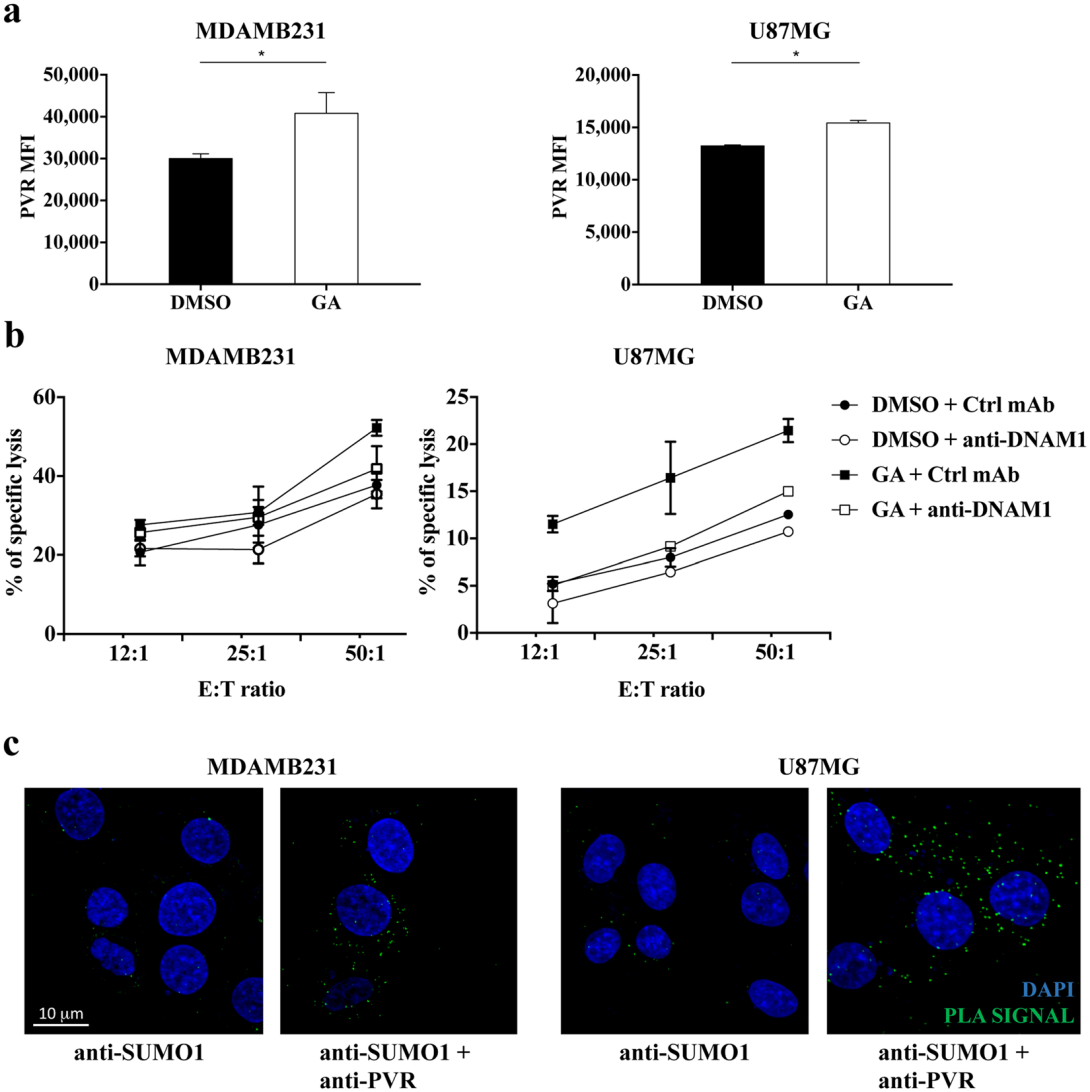
PVR expression is regulated by SUMO pathway in breast cancer and glioma cell lines. (**a**) MDAMB231 and U87MG cells were treated overnight with 25 μg/mL of GA or vehicle alone (DMSO), and then evaluated for PVR surface expression by immunofluorescence and FACS analysis with FACSCanto (BD Bioscience). Means ±SD from three independent experiments are shown. *p < 0.05, Student T test. (**b**) Primary cultured NK cells were pre-treated for 20 minutes at RT with anti-DNAM1 neutralizing mAb or with anti-CD56 (Ctrl mAb) and were used as effector cells in a 4 hours ^51^Cr release assay toward GA or vehicle (DMSO)-treated MDAMB231 and U87MG cells. (**c**) PLA was performed on MDAMB231 breast cancer cells and U87MG glioma cells as in Fig. 4c. Images are representative of three independent experiments, and were acquired with zoom 2 using 60X/1.35NA oil immersion objective. Z-projections of 30 slices are shown.

We focused our attention on MDAMB231 breast cancer and U87MG glioma cell lines, and we first analysed whether the SUMO pathway regulates PVR surface expression. Despite a high basal level of ligand expression, the GA treatment resulted in a slight but reproducible up-regulation of PVR membrane expression on both cell lines (Fig. 8a) and rendered them more susceptible to DNAM1-mediated NK cell cytotoxicity (Fig. 8b). We have then evaluated PVR SUMOylation status by performing PLA reaction. Fluorescent spots corresponding to PVR-SUMO interaction only appeared in the presence of anti-SUMO1 and anti-PVR specific antibodies and not in negative control reactions (Fig. 8c), indicating that PVR undergoes SUMOylation.

These results demonstrate that PVR expression can be regulated by the SUMO pathway also in tumors other than MM, and suggest that PVR SUMOylation represents a more general strategy to prevent DNAM1-dependent tumor cell recognition and killing by NK cells.

## Discussion

MM is a still incurable neoplastic disease and represents the 13% of all haematological malignancies^10, 11^. Among currently used therapeutic strategies, immunomodulatory drugs have recently been proven to successfully potentiate the ability of immune cells, including NK cells, to fight tumor spreading^36, 37^. During tumor progression, MM cells produce immune suppressive factors and reduce surface expression of ligands for NK cell activating receptors^12, 38^, thus rendering NK cells progressively unable to efficiently counteract malignant PC expansion^4–6^. In this context, it is still currently unknown whether post-translational modifications regulate the expression of these ligands on MM cells thus affecting their susceptibility to NK cell-mediated killing.

Here, we show that the surface expression of a ligand for the activating receptor DNAM1, PVR, is regulated at post-translational level in MM cells. In particular, our major findings were: (1) PVR undergoes SUMOylation in MM cells; (2) SUMOylated PVR is retained intracellularly; (3) SUMOylation of PVR, by preventing its surface expression, impairs NK cell-mediated MM surveillance.

SUMO conjugation to protein substrates is frequently up-regulated during tumor transformation, and the function of SUMOylated proteins has been extensively studied in the context of nuclear-associated processes, such as transcriptional regulation, chromatin remodelling, DNA repair, telomere maintenance, nucleo-cytoplasmic transport, and mitosis^29, 30^. More recently, a role for SUMOylation in the regulation of membrane protein expression and/or function was also becoming clear. SUMOylation regulates the expression and function of glucose transporters^39^ and the expression of the mutated cystic fibrosis transmembrane conductance regulator in cystic fibrosis^40^. Moreover, in the case of kainate receptors, the voltage-gated potassium channels and the transforming growth factor β receptor (TGFβR), SUMO conjugation directly regulates receptor activity^41–43^. Notably, in neurons, SUMOylation of the kainate receptor subunit GluR6 increases ligand-induced receptor endocytosis^42^.

Our findings provide the first evidence of a ligand of NK cell activating receptors that is regulated by the SUMO pathway.

Indeed, we demonstrate that PVR is subjected to SUMOylation in MM cells likely as a consequence of the up-regulation of the SUMO pathway that occurs during transformation^28^. Although we were unable to quantify the amount of PVR that is modified by SUMO, it is well established that SUMO modification of a small substrate population is sufficient to elicit a functional consequence^29^.

Concurrently, we found that PVR is preferentially located in intracellular compartments both in MM cell lines and PCs derived from MM patients, and that inhibition of the SUMO pathway promotes PVR translocation to the cell surface. These observations strongly suggest that SUMOylation of PVR is responsible for its intracellular localization contributing to tumor evasion. Whether SUMOylation of PVR prevents its membrane localization or promotes its constitutive internalization is currently unclear.

We observed that the PVR intracellular localization as well as protein surface expression in PCs derived from MM patients is independent from the clinical stage. This result is compatible with the murine model of MM in which PVR expression does not change during MM progression^17^. We speculate that during tumor transformation stressful events such as DNA damage, in addition to promote transcriptional PVR expression^14, 22–25^, up-regulate the SUMO pathway that in turn prevents surface PVR expression during MM progression.

Along with PVR, we found that the other DNAM1 ligand, Nectin2, is also prevalently expressed as intracellular pool. However, Nectin2 surface expression is not regulated by SUMO pathway, suggesting the involvement of post-translational modifications different than SUMOylation. To this regard, our preliminary observations indicate that Nectin2 undergoes ubiquitination (Zitti *et al*. unpublished observations). Thus, it is likely that more than one post-translational modification could cooperate to regulate DNAM1 ligand surface expression. Experiments are currently ongoing to further explore this possibility.

The functional consequence of the increased PVR surface expression is the improvement of MM susceptibility to NK cell-mediated killing. Even though we found that the SUMO pathway regulates PVR but not Nectin2 expression, it is likely that the up-regulation of PVR expression is sufficient to affect MM susceptibility to NK cell-mediated lysis. Indeed, PVR was found to bind to DNAM1 with a higher affinity than Nectin2^44^.

Although an indispensable role for DNAM1 is envisaged by its contribution to NK cell-mediated MM immune surveillance *in vivo* ^17^, we cannot rule out that the expression of NK cell activating ligands different than PVR can be also modulated by SUMO pathway.

Regarding receptors that are implicated in MM cell recognition^12–14^, we have excluded the contribution of NKG2D/NKG2D ligand axis in our experimental setting. Of note, the requirement of DNAM1-PVR interaction appears to be particularly relevant when tumor cells lack the expression of other NK cell-activating ligands^45^, as happens in advanced MM stages during which NKG2D ligands are down-modulated^12, 38^.

In addition, we have also excluded (data not shown) the involvement of the SUMO pathway in the surface expression regulation of CS1 (CD2 subset 1), a surface molecule of the SLAM family highly expressed on MM cells that represents a promising target for the treatment of MM^46^.

Several reports have demonstrated transcriptional induction of DNAM1 ligands in tumor cells including MM, and some of the transcription factors involved have been recently identified^22–25, 37^.

Our data add a new level of complexity, supporting a role for post-translational mechanisms in the regulation of PVR expression. Indeed, we demonstrate that PVR itself undergoes SUMOylation and that this pathway regulates PVR expression without affecting its mRNA and protein levels.

Previous results demonstrated that chemotherapeutic drugs including melphalan, doxorubicin and bortezomib, increase DNAM1 ligand expression on MM cells^14, 47^ acting at transcriptional level through the activation of different signaling pathways. Whether those chemotherapeutic drugs can also affect PVR expression through the regulation of the SUMO pathway is currently unknown.

It is interesting to point out that we have provided results demonstrating that the SUMO pathway regulates PVR expression in tumors other than MM, suggesting that PVR SUMOylation is a more general strategy to prevent DNAM1-dependent recognition and killing by NK cells.

A post-translational mechanism to repress surface expression of DNAM1 ligands was also reported in hepatocellular carcinoma^48^ and upon Cytomegalovirus (CMV) infection^49, 50^. The activation of Unfolded Protein Response (UPR) resulted in down-regulation of PVR surface expression by protein degradation in hepatocellular carcinoma, while upon CMV infection an immature form of PVR is retained in ER by the viral protein UL141. The same protein is also responsible for Nectin2 constitutive degradation^50^. These results suggest that post-translational regulation of DNAM1 ligand expression represents a common mechanism to evade NK cell recognition both in transformed and infected cells.

In line with these results, the expression of the murine ligand for NKG2D receptor Mult1 is prevented by a constitutive protein ubiquitination and degradation^51^, suggesting that post-translational modifications can be more widely involved in the regulation of NK cell activating ligands.

In the context of the innate immune response, a post-translational regulation of ligands for activating NK cell receptors could contribute to modulate very rapidly target cell susceptibility to NK cell recognition.

Of note, DNAM1 and NKG2D are not exclusively expressed on NK cells but are shared by other lymphocytes^52, 53^. Thus, post-translational modifications of their ligands, by preventing target cell recognition, may have a broader impact on anti-tumor immunity.

Collectively, our results shed light into the molecular mechanisms that regulate immune activating ligand expression. We reveal a previously undefined role for the SUMO pathway that could represent an attractive candidate for therapeutic intervention aimed at facilitating immune cell-mediated recognition and killing of tumor cells.

## Methods

### Cell lines and clinical samples

The human MM cell lines SKO-007 (J3), U266, ARK, OPM-2, RPMI, LP1, and ARP were kindly provided by Prof. P. Trivedi (“Sapienza” University of Rome). The human breast cancer cell line MDA-MB-231 and the human glioma cell line U87MG were purchased by ATCC.

Cell line identity was verified by morphology, growth, immunophenotypic characteristics and biologic behaviour according to the provider recommendations, and periodically tested for mycoplasma contamination by EZ-PCR Mycoplasma Test Kit (Biological Industries, 20–700–20). All cell lines were kept in culture for less than two consecutive months. The cell lines were maintained at 37 °C in 5% CO_2_ in RPMI 1640 (Life Technologies) supplemented with 10% Fetal Calf Serum (FCS).

Primary cultured NK cells were obtained from 10-day co-cultures of PBMCs (4 × 10^5^ cells/mL) with the irradiated EBV-transformed B-cell line RPMI 8866 (10^5^ cells/mL) at 37 °C in 5% CO_2_ in the absence of IL-2, as previously described^54^.

Peripheral blood and bone marrow samples from patients with MM were managed at the Division of Hematology (“Sapienza” University of Rome). The study was conducted according to protocols approved by the Ethics Committee of “Sapienza” University of Rome (Rif. 3373) and informed consent, in accordance with the Declaration of Helsinki, was obtained from all patients.

MM bone marrow aspirates were cleared from red cells in a buffer containing 1.5 mol/L NH_4_Cl, 100 mmol/L NaHCO_3_ and 10 mmol/L ethylenediaminetetraacetic acid (EDTA). Bone marrow-derived mononuclear cells were maintained at 37 °C and 5% CO_2_ in complete medium supplemented with 20 ng/mL human recombinant IL-3 (Pepro-Tech, 200–03) and 2 ng/mL human recombinant IL-6 (Pepro-Tech, 200–06), as previously described^55^.

In some experiments MM cells were purified using anti-CD138 magnetic beads (Miltenyi Biotec, 130–051–301). More than 95% of purified cells expressed CD38 and CD138.

For experiments in which the SUMO pathway was inhibited, MM cells were cultured over-night at 37 °C in 5% CO_2_ at the concentration of 2 × 10^6^ cells/mL with different doses of Ginkgolic Acid (Calbiochem, 345887) or equal amounts of DMSO. A dose-response curve was constructed to identify the doses that did not affect cell viability and the dose of 25 μg/mL was chosen.

### Virus production and *in vitro* transduction

pLKO.1-puro lentiviral vector expressing a short hairpin RNA targeting UBC9 was kindly provided by Dr. Shih (Academia Sinica, Taipei, Taiwan). The nontargeting control shRNA vector (pLKO.1) was purchased from Sigma-Aldrich. For lentivirus production, the Phoenix packaging cell line HEK293 was transfected with 5 μg of viral DNA (pLKO.1 or pLKO.1-shUBC9) together with pVSVG and psPAX2 packaging vectors, using Lipofectamine 3000 (Life Technologies, L3000001). After transfection, cells were plated in fresh medium, and 48 hours later virus-containing supernatants were harvested, filtered and used immediately for two cycles of infections as follows: 2 mL viral supernatant in complete medium containing 8 μg/ml Polybrene (Sigma-Aldrich, 107689) were used to infect 1 × 10^6^ ARK or OPM2 cells for 2 hours. After 24 hours from the infection cells were selected with 1 μg/mL puromycin (Sigma Aldrich, P8833).

### Immunofluorescence and FACS analysis

The identification of malignant PCs derived from bone marrow aspirates was performed by means of CD38-PE or CD38-APC (BD Biosciences, HIT2) and CD138-FITC (BD Biosciences, HI15). Surface ligands expression on patient-derived PCs was evaluated by means of PE-conjugated anti-PVR mAb (Biolegend, SKII.4), and APC-conjugated anti-Nectin2 mAb (R&D Systems, FAB2229A) after gating on the CD38^+^ CD138^+^ PC population. Cells were fixed and permeabilized with Fixation/Permeabilization kit (BD Biosciences) according to manufacturer’s instructions to evaluate total cellular proteins. The surface expression of DNAM1 ligands on MM cell lines was analysed by immunofluorescence staining using unconjugated anti-PVR mAb (SKII.4, kindly provided by Prof. M. Colonna, Washington University, St. Louis, MO) followed by APC-conjugated goat anti-mouse (Jackson ImmunoResearch Laboratories, GAM-APC IgG), and APC-conjugated anti-Nectin2 (R&D Systems, FAB2229A). To evaluate total cellular proteins, MM cells were fixed with 2% paraformaldehyde (Sigma Aldrich, 158127) for 20 minutes at room temperature, permeabilized with 0.1% saponin (Sigma Aldrich, S7900) for 30 minutes at 4 °C, and washed with PBS 0.5% BSA before the staining. Samples were acquired using a FACSCalibur (BD Biosciences) in data shown in Figs 1b, 2b and c and Supplementary Figure S2, and FACSCanto (BD Biosciences) in all the other experiments.

The mean fluorescence intensity (MFI) subtracted from the MFI of the isotype control antibody was measured using FlowJo software (Ashland, OR).

### Confocal microscopy and Proximity ligation assay (PLA)

ARK cells were plated on poly-L-lysine-coated multichamber slides (LabTek, Thermo Scientific) and let adhere for 30 minutes at 37 °C. Cells were then fixed and permeabilized, as previously described^54^.

In experiments in which PVR expression was analysed cells were stained with the anti-PVR mAb (ThermoFisher Scientific, D171) followed by the AlexaFluor 488-conjugated goat anti-mouse IgG (Life Technologies). After extensive washing cells were counterstained with DAPI (Life Technologies, D1306) and coverslips were mounted using SlowFade gold reagent (Life Technologies, S36936).

Proximity Ligation Assay was performed using Duolink PLA *In Situ* Green Starter Kit (SigmaAldrich, Mouse/Rabbit) according to manufacture’s instructions. Anti-PVR (ThermoFisher Scientific, D171) and anti-SUMO1 (Enzo Life Sciences, BML-PW9460) were used as primary Abs.

High-resolution images (800 × 800 pixel, 8 μs/pixel) were acquired at room temperature using IX83 FV1200 MPE laser-scanning confocal microscope with a 60 × /1.35 NA UPlanSAPO oil immersion objective (all from Olympus) as previously described^56^. Images were processed with Fiji ImageJ software. Were indicated, image stacks (40 slices of 0.5 μm z-step size) were acquired. Data quantification was performed with FiJi ImageJ software and 3D reconstructions were obtained with Imaris software v.8.1.2 (Bitplane).

### RNA isolation, RT-PCR and Real Time PCR

Total RNA was extracted using TRIzol (Life Technologies, AM9738), according to manufacturer’s instructions. Light absorbance at 260 nm (A_260_) and the ratio of A_260_/A_280_ were measured to determine the concentration and quality of the extracted total RNA, respectively. One microgram of total RNA was used for cDNA first strand synthesis in a 25 μL reaction volume according to the manufacturer’s protocol for Moloney murine leukemia virus reverse transcriptase (Promega). Real-Time PCR was performed using the ABI Prism 7900 Sequence Detection system (Applied Biosystems). cDNAs were amplified in triplicate with primers for PVR (Hs00197846_m1) and GAPDH (Hs99999905_m1) both conjugated with fluorocrome FAM (Applied Biosystems). The level of ligand expression was measured using threshold cycle (Ct). The Ct was obtained by subtracting the Ct value of the gene of interest (PVR) from that of housekeeping gene (GAPDH). In the present study we used the Ct of the untreated sample or the mock-transfected one as the calibrator. The fold change was calculated as 2^−ΔΔCt^, where ΔΔCt is the difference between the Ct of the sample and the Ct of the calibrator (according to the formula the value of the calibrator in each run is 1). The analysis was performed using SDS version 2.2 software (Applied Biosystem).

### Immunoprecipitation and Western blot analysis

Cells were lysed in a buffer containing 1% Triton X-100, 0,1% SDS, 50 mmol/L Tris-HCl pH 7.4, 150 mmol/L NaCl, 0,5% sodium deoxycholate, 1 mmol/L EGTA, 1 mmol/L EDTA, 5 mmol/L MgCl_2_, 1 mmol/L PMSF, 1 mmol/L Na_3_VO_4_, 5 mmol/L NaF pH 8, 20 mmol/L N-ethyl-malemide, 10 μg/mL of aprotinin and 5 μg/mL leupeptin, incubated 30 min on ice and then centrifugated at 13000 X g for 30 minutes at 4 °C and the supernatant was collected as whole-cell extract. Bio-Rad Protein Assay was used to determine protein concentration.

Immunoprecipitations was performed as previously described^57^ using anti-PVR (SKII.4) or control mouse IgG (Santa Cruz Biotechnology). Immunoprecipitates or total cell lysates were resolved by SDS-polyacrylamide gel (PAGE), proteins were then electro-blotted onto nitrocellulose membranes (Schleicher & Schuell, GEH10600002), and western blotting was performed as previously described^57^ with the following Abs: anti-PVR (S-18, Santa Cruz Biotechnology), anti-SUMO1 (Abcam, Y299), anti-UBC9 (BD Biosciences, 50/ubc9), anti-β actin (Sigma-Aldrich, AC-74). Fiji Image J software was used to perform densitometric analysis.

### *In vitro* SUMOylation assay

A recombinant full-lenght human form of PVR (Abnova, H00005817) was used as substrate for *in vitro* SUMOylation. The reaction was carried out using 200 ng of the target protein in a 20 μL reaction volume containing E1 and E2 enzymes involved in substrate SUMOylation, SUMO 1/2/3 proteins and SUMOylation buffer for 1 hour at 37 °C in the presence of ATP, according to manufacturer’s instructions (Enzo Life Sciences). Negative control reaction omitting Mg-ATP cofactors (required for E1 activation) was performed. After *in vitro* SUMOylation samples were eluted with SDS-sample buffer and resolved by SDS/PAGE.

### ^51^Cr-release assay, degranulation assay, and conjugation assay

Primary cultured NK cells were pretreated with anti-DNAM1 (Serotec, DX11) or anti-CD56 (Serotec, NCAM16.2) Abs at 1 μg/10^6^ cells for 20 minutes at room temperature and employed as effectors cells in cytotoxicity or degranulation assays using as cell targets UBC9-silenced or mock-transfected ARK cells.

The ^51^Cr-release assay (4 hours) was used to measure cytotoxic activity against ARK target cells, as previously described^54^. Maximum release was assessed by incubating ^51^Cr-labeled target cells with 2.5% SDS, while spontaneous release was evaluated by incubating the target cells alone in culture medium. The percentage of specific lysis was determined as follows: ((mean cpm experimental release – mean cpm spontaneous release)/(mean cpm maximal release - mean cpm spontaneous release)) × 100. Lytic Units for 1 × 10^6^ effector cells were calculated as previously described^54^.

Degranulation was determined by cell-surface expression of the lysosomal marker CD107a. Primary cultured NK cells and ARK cells were co-cultured at 2:1 E:T ratio in complete medium for 4 hours at 37 °C in the presence of CD107a-APC mAb (BD Biosciences, H4A3). After 1 hour, 50 μmol/L monensin (Sigma Aldrich, catalog number) was added. Cells were then stained with CD56-PE (BD Biosciences, NCAM16.2), CD3-FITC (Biolegend, OKT3) and CD138-PerCP-CY5.5 (BD Biosciences, MI15) to gate the CD56^+^ CD3^−^ CD138^−^ NK cell population and the analysis was performed with FACSCanto (BD Biosciences).

Degranulation of patient-derived NK cells was performed as previously described^58^ with some modifications. Briefly, as source of effector cells bone marrow purified NK cells cultured overnight with 200 U/mL IL-2 were used. Autologous patient-derived PC treated with GA or vehicle alone were used as target cells and were incubated with effector cells at an E:T ratio of 2:1 in complete medium at 37 °C for 2 hours. At the end of the co-culture cells were washed with PBS 2% FCS 5 mmol/L EDTA, and stained with CD107a-APC, CD56-PE, CD3-FITC and CD138-PerCP-CY5.5 to gate the CD56^+^CD3^−^CD138^−^ NK cell population. Analysis was performed using a FACSCanto (BD Biosciences).

For the conjugation assay primary cultured NK cells were labelled with CellTracker DeepRed (Life Technologies, C34565) while Mock or UBC9 silenced ARK cells were labelled with 2.5 μmol/L CFSE (Sigma Aldrich, 21888). Effector and target cells were spun at 100 g and left to interact for 30 minutes at 1:2 E:T ratio.

The percentage of conjugates was evaluated by FACSCanto (BD Biosciences) and calculated on red positive gated cells as follows: (red and green doublets/total red cells)x100.

### Statistical analysis

Statistical significance between two groups was determined by performing two-tailed, paired Student’s t-test. Differences between multiple groups were analysed with two-way analysis of variance (ANOVA) using Šidák method for multiple comparisons. The non-parametric Wilcoxon matched-pairs signed rank test was used to analyse data coming from groups of patients. Prism 6 (GraphPad) software was used. Graphs show mean values, and all error bars represent the SD.

### Data availability

The datasets generated during and/or analysed during the current study are available from the corresponding author on reasonable request.

## Electronic supplementary material

Supplementary multimedia file

Supplementary Information
