# Supplementary material for: Innate immune activating ligand SUMOylation affects tumor cell recognition by NK cells
Source: Sci Rep. 2017 Sep 5;7:10445. doi: 10.1038/s41598-017-10403-0 (PMC5585267; doi:10.1038/s41598-017-10403-0)
Supplement: Supplementary file 2 — Supplementary Information [file 41598_2017_10403_MOESM2_ESM.doc]

**Innate immune activating ligand SUMOylation affects tumor cell recognition by NK cells**

Beatrice Zitti^1^**^+^**, Rosa Molfetta^1^**^+^**^*^, Cinzia Fionda^1^, Linda Quatrini^1§^, Helena Stabile^1^, Mario Lecce^1^, Valeria de Turris^2^, Maria Rosaria Ricciardi^3^, Maria Teresa Petrucci^4^, Marco Cippitelli^1^, Angela Gismondi^1^, Angela Santoni^1,5^ and Rossella Paolini^1*^

^1^Department of Molecular Medicine, “Sapienza” University of Rome, Laboratory affiliated to Istituto Pasteur Italia - Fondazione Cenci Bolognetti, “Viale Regina Elena 291, 00161, Rome, Italy.

^2^ Center for Life Nanoscience, Istituto Italiano di Tecnologia, Rome, Italy.

^3^Division of Hematology, Department of Clinical and Molecular Medicine, Sapienza University of Rome, Rome, Italy.

^4^Department of Cellular Biotechnologies and Hematology, Sapienza University of Rome, Rome, Italy.

^5^Istituto Mediterraneo di Neuroscienze, Neuromed, Pozzilli, Italy.

^§^Present address: Centre d’Immunologie de Marseille-Luminy, Aix Marseille Université UM2, Inserm, U1104, CNRS UMR7280, 13288 Marseille, France

^+^B. Zitti and R. Molfetta contributed equally to this work; A. Santoni and R. Paolini share senior co-authorship.

*****Correspondence and requests for materials should be addressed to RP and RM (email: [rossella.paolini@uniroma1.it; rosa.molfetta@uniroma1.it](mailto:rossella.paolini@uniroma1.it;rosa.molfetta@uniroma1.it) )

**Supplementary Table 1: Patients characteristics.**

| **Number** | **Clinical Stage** | **Isotype** | **% PCs in BM** |
| --- | --- | --- | --- |
| **1** | Relapse | IgA-k | 11 |
| **2** | Relapse | IgG-k | 19 |
| **3** | MGUS | IgG-L | 2 |
| **4** | Relapse | micro k | 54 |
| **5** | MGUS | IgG-k | 7 |
| **6** | MGUS | IgA-L | 5 |
| **7** | Smoldering | IgA-L | 2 |
| **8** | Onset | IgG-k | 52 |
| **9** | Relapse | IgG-L | 29 |
| **10** | Relapse | IgG-k | 32 |
| **11** | Relapse | IgG-k | 13 |
| **12** | Onset | IgA-k | 30 |
| **13** | Onset | IgG-L | 31 |
| **14** | Onset | IgG-k | 42 |
| **15** | Onset | IgG-k | 28 |
| **16** | Relapse | IgG-L | 19 |
| **17** | Smoldering | IgG-L | 26 |
| **18** | MGUS | IgG-L | 16 |
| **19** | MGUS | IgG-k | 7 |
| **20** | Smoldering | IgG-L | 13 |
| **21** | MGUS | IgA-k | 9 |
| **22** | Smoldering | IgG-L | 22 |
| **23** | Smoldering | IgG-L | 0.4 |
| **24** | MGUS | IgG-k | 3 |
| **25** | Smoldering | IgG-k | 17 |
| **26** | Relapse | IgA-k | 34 |
| **27** | Onset | IgG-L | 60 |
| **28** | Smoldering | IgA-L | 34 |
| **29** | MGUS | IgG-L | 9 |
| **30** | Smoldering | IgG-L | 12 |
| **31** | Onset | IgG-k | 3.6 |
| **32** | Relapse | IgG-k | 58 |
| **33** | Smoldering | IgA-k | 48 |
| **34** | Relapse | IgG-L | 37 |
| **35** | Smoldering | IgA-L | 22 |
| **36** | Relapse | IgG-L | 71 |
| **37** | Onset | IgG-k | 60 |
| **38** | Relapse | IgG-k | 32 |

Patients were classified according to Durie and Salmon’s staging system.

MM evolves from a pre-neoplastic disorder called *“*Monoclonal Gammopathy of Undetermined Significance*”* (MGUS), through phases of asymptomatic (Smoldering) and syntomatic (Onset) disease.

**
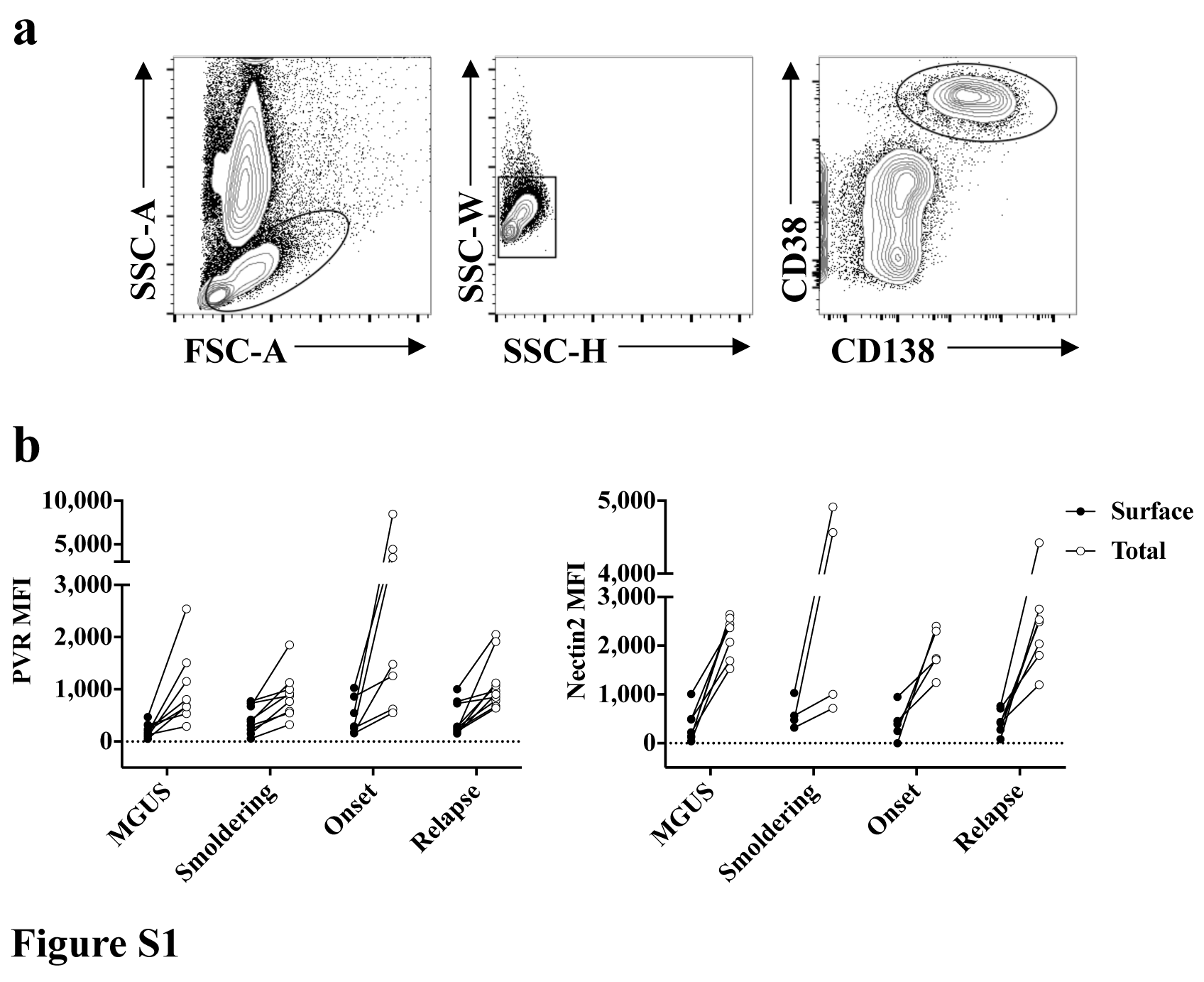
**

**Supplementary Figure S1**

**a**: Malignant PCs were first gated based on morphological characteristics (left panel) and then doublets were excluded (middle panel) before gating on CD138/CD38 double positive cells.

**b**: PVR (left panel) and Nectin2 (right panel) surface and total (surface plus intracellular) expression was analysed during disease progression, and patients were divided according to the stage of transformation. Each dot represents single patient.

**
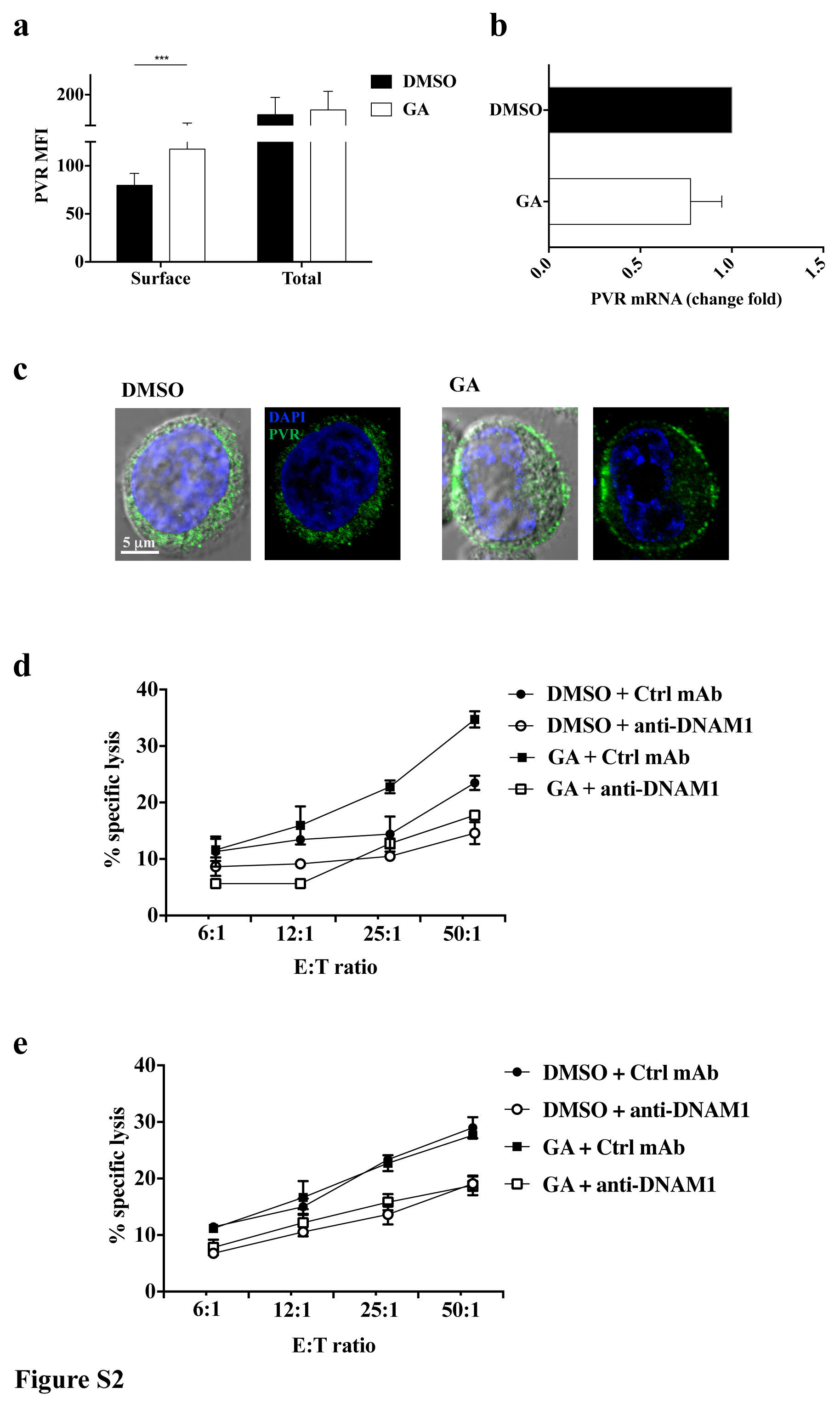
**

**Supplementary Figure S2**

**a**: PVR expression on ARK cells upon GA treatment was evaluated as in Fig. 1b. Data represent the means ± SD of three independent experiments. ** p< 0.005, Two-way RM ANOVA.

**b**: Total RNA, extracted from ARK cells treated with GA or vehicle alone (DMSO) was used for cDNA first-strand synthesis, and Real-time polymerase chain reaction for PVR mRNA was performed as in Fig. 5b. Relative mRNA amount, normalized with GAPDH, was expressed as arbitrary units and referred to mock-transfected cells, considered as calibrator.

Means ± SD of three independent experiments are shown.

**c**: ARK cells treated as in b were stained with anti-PVR mAb (clone D171) followed by Alexa 488-conjugated goat anti-mouse Ab and counterstained with DAPI. Fluorescence and Differential Interference Contrast images were acquired with zoom3 using 60X/1.35NA oil immersion objective. Single optical slices are shown.

**d-e**: Primary cultured NK cells were pretreated for 20 minutes at RT with anti-DNAM1 neutralizing mAb or with anti-CD56 (Ctrl mAb) and were used as effector cells in a 4 hours ^51^Cr release assay toward GA or DMSO treated ARK (d) or LP1 (e) cells. The percentage of specific lysis from three independent experiments (mean ± SD) is shown.

**
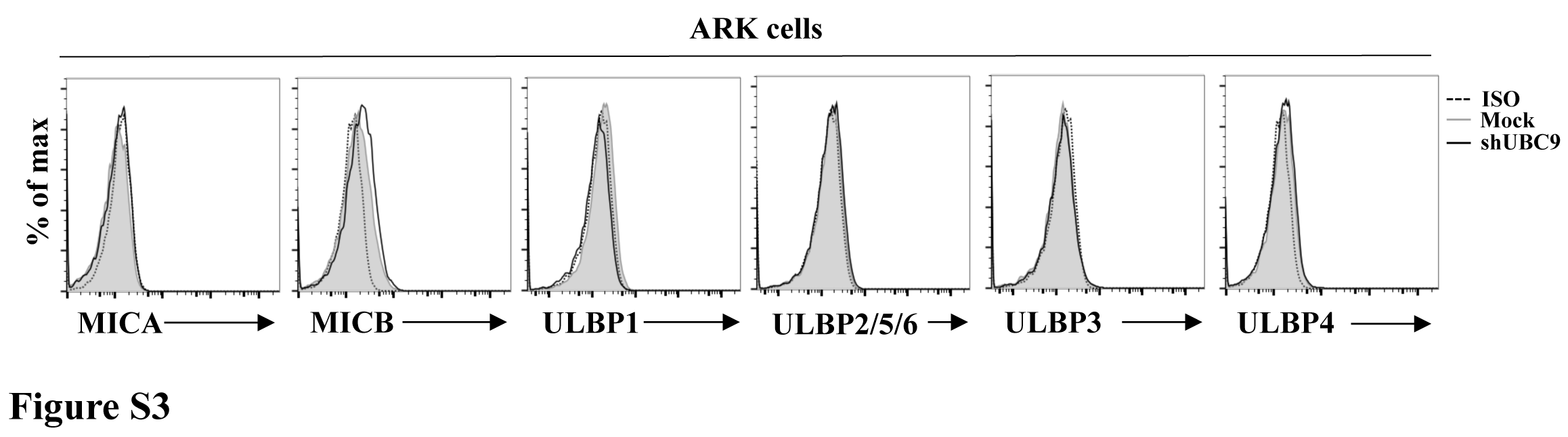
**

**Supplementary Figure S3**

NKG2D ligand expression evaluated on UBC9-silenced or Mock transfected ARK cells by immunofluorescence and FACS analysis using the following antibodies: anti-MICA (MAB159227), anti-MICB (MAB236511), anti-ULBP1 (MAB170818), anti-ULBP2 (MAB165903) and anti-ULBP3 (MAB166510), all from R&D Systems (Minneapolis, MN).

**Supplementary Multimedia File 1**

Animation of 3D reconstruction of all optical slices (n=40) acquired along z-axis.
